# Emerging Biomarkers in the Era of Personalized Cancer Medicine

**DOI:** 10.1155/2019/5907238

**Published:** 2019-08-14

**Authors:** Biagio Ricciuti, Giulia C. Leonardi, Marta Brambilla

**Affiliations:** ^1^Thoracic Oncology Unit, University of Perugia, Perugia, Italy; ^2^Department of Biomedical and Biotechnological Sciences, Pathology and Oncology Section, University of Catania, Catania, Italy; ^3^Istituto Nazionale dei Tumori (INT), Milan, Italy

Personalized oncology is an evidence-based, patient's tailored approach that is aimed at identifying and treating each cancer patient based on its genetic makeup and molecular features. Biomarkers are a key player in personalized cancer medicine, and biomarker discovery and development represent an area of active research and specific challenges. Currently, predictive and prognostic biomarkers that can guide the therapeutic decision-making process are already available in the clinical practice. Patients with solid tumors and hematological malignancies derive great clinical benefit and access to specific treatment upon specific biomarker assessment. First, the advent of molecular diagnostics with single/multi-, gene/protein variant detection enabled the identification of patients with exquisite sensitivity to targeted therapies or immunotherapy. More recently, the advent of high-throughput genomic and molecular profiling and “omics” techniques has led to the discovery of a wide spectrum of potentially relevant biomarkers that will hopefully provide a deeper understanding of cancer biology and host interaction, raising the bar of personalized cancer medicine. This special issue includes selected articles focusing on emerging biomarkers and their potential clinical application in different solid tumors.

T. Shen et al. showed that kinesin family member 20A (KIF20A) promotes the growth of the bladder tumors *in vivo*, and its overexpression associates with a poor prognosis in patients with bladder cancer. This study suggests that KIF20A may become an independent prognostic factor in patients with bladder cancer and a potential therapeutic target as selective KIF20A inhibitors are in development.

C.-Y. Huang and colleagues demonstrated that GRP94 silencing may increase the resistance of osteosarcoma cell lines (MG63 and 143B) to paclitaxel, gemcitabine, and epirubicin treatments by inhibiting the induction of apoptosis, suggesting that GRP94 may be a key biomarker for the chemotherapeutic response of osteosarcoma.

By using gynecologic cancer cell lines with known TP53 mutational status, X. Meng et al. demonstrated that proteasome inhibition induced cell death in cells with two recurrent gain of function (GOF) TP53 mutations (R175H and R248Q) and that the addition of a histone deacetylase inhibitor (HDACi) enhanced this effect. This study provides preliminary evidence for a novel therapeutic strategy for tumors with GOF TP53 mutations using drugs that are already being advanced in clinical trials.

C. Mecca and colleagues analyzed the rationale of targeting mTOR in GBM and the available preclinical and clinical evidences supporting the choice of this therapeutic approach, highlighting the different roles of mTORC1 and mTORC2 in GBM biology.

G. Cervino and colleagues provided an overview of the emerging diagnostic and prognostic biomarkers in oral cancer, which still represents one of the leading causes of death in developing countries.

In conclusion, this special issue wants to emphasize the central role of biomarker identification and implementation as the cornerstone of personalized cancer medicine and highlights how the bench to bedside translational science has a great impact on the clinical practice and patient's quality of life.

